# LptD depletion disrupts morphological homeostasis and upregulates carbohydrate metabolism in *Escherichia coli*

**DOI:** 10.1093/femsmc/xtad013

**Published:** 2023-08-10

**Authors:** Frida Svanberg Frisinger, Bimal Jana, Juan C Ortiz-Marquez, Tim van Opijnen, Stefano Donadio, Luca Guardabassi

**Affiliations:** Department of Veterinary and Animal Sciences, Faculty of Health and Medical Sciences, University of Copenhagen, 1870 Frederiksberg, Denmark; Department of Veterinary and Animal Sciences, Faculty of Health and Medical Sciences, University of Copenhagen, 1870 Frederiksberg, Denmark; Biology Department, Boston College, Chestnut Hill, MA 02467, United States; Biology Department, Boston College, Chestnut Hill, MA 02467, United States; Biology Department, Boston College, Chestnut Hill, MA 02467, United States; Naicons Srl, Milan, 20139, Italy; Department of Veterinary and Animal Sciences, Faculty of Health and Medical Sciences, University of Copenhagen, 1870 Frederiksberg, Denmark

**Keywords:** Escherichia coli, LptD, carbohydrate metabolism, colistin, colanic acid

## Abstract

In a previous *in silico* study, we identified an essential outer membrane protein (LptD) as an attractive target for development of novel antibiotics. Here, we characterized the effects of LptD depletion on *Escherichia coli* physiology and morphology. An *E. coli* CRISPR interference (CRISPRi) strain was constructed to allow control of *lptD* expression. Induction of the CRISPRi system led to ∼440-fold reduction of gene expression. Dose-dependent growth inhibition was observed, where strong knockdown effectively inhibited initial growth but partial knockdown exhibited maximum overall killing after 24 h. LptD depletion led to morphological changes where cells exhibited long, filamentous cell shapes and cytoplasmic accumulation of lipopolysaccharide (LPS). Transcriptional profiling by RNA-Seq showed that LptD knockdown led to upregulation of carbohydrate metabolism, especially in the colanic acid biosynthesis pathway. This pathway was further overexpressed in the presence of sublethal concentrations of colistin, an antibiotic targeting LPS, indicating a specific transcriptional response to this synergistic envelope damage. Additionally, exposure to colistin during LptD depletion resulted in downregulation of pathways related to motility and chemotaxis, two important virulence traits. Altogether, these results show that LptD depletion (i) affects *E. coli* survival, (ii) upregulates carbohydrate metabolism, and (iii) synergizes with the antimicrobial activity of colistin.

## Teaser

LptD depletion leads to morphological changes and upregulates carbohydrate metabolism in *Escherichia coli*.

## Introduction

The outer membrane (OM) is a distinguishing feature in Gram-negative bacteria. It acts as a selective permeability barrier, preventing intracellular access to many noxious substances found in the extracellular milieu, including antibiotics (Delcour 2009). This hallmark organelle plays a pivotal role in cell survival and virulence in Gram-negative bacteria but is absent in Gram-positive bacteria, including many taxa that reside in the gut microbiota and which have beneficial effects on host health. The OM is therefore an interesting target for developing novel antibiotics that selectively interfere with Gram-negative opportunistic pathogens inhabiting the intestinal tract, like *Escherichia coli*, while leaving the surrounding Gram-positive bacterial community undisturbed and thus reducing the risk of dysbiosis.

OM biogenesis is carried out by three systems: the lipoprotein transport system (LOL), LPS transport system (LPT), and the β-barrel assembly machinery (BAM) (Konovalova et al. 2017). Here, we focus on the LPT pathway, which is responsible for transporting intracellularly produced LPS across the periplasmic space to the cell surface. In *E. coli*, the pathway consists of seven proteins encoded by the essential genes *lptA, lptB, lptC, lptD, lptE, lptF*, and *lptG*. In this pathway, the LptB_2_FG ABC transporter, which is strongly associated with LptC, is responsible for extracting the LPS from the inner membrane (IM). LptC is associated with LptA, forming a periplasmic bridge, ultimately making contact with LptD, a β-barrel protein that, in complex with LptE, is responsible for inserting the LPS into the OM outer leaflet (Sperandeo et al. 2019).

**Figure 1.**
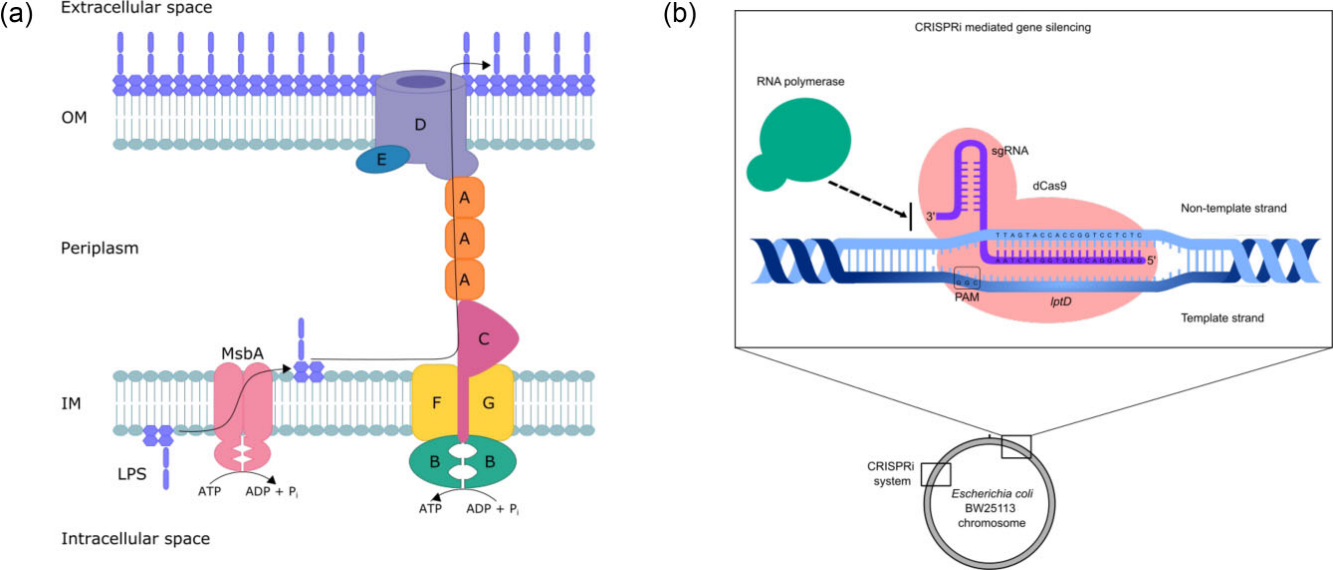
Gene silencing of LptD in *E. coli* using CRISPRi. **(a)** Schematic of the Lpt system in *E. coli*. LPS is extracted across the IM by LptB2FG together with MsbA and transported through the periplasm by the transenvelope bridge formed by LptA to finally be exported to the OM outer leaflet by LptDE. **(b)** Schematic of the Mobile-CRISPRi system. The system consists of a chromosomally integrated dCas9 and an sgRNA. The catalytically inactive dCas9 (pink) is coupled to an sgRNA (purple), which targets the complex to a specific location on the target lptD gene and acts as a physical barrier to RNA polymerase (green), sterically hindering transcription of the gene.

Using an *in silico* approach, we previously identified LptD as a promising novel antimicrobial drug target selective for *E. coli* and *Klebsiella pneumoniae* (Svanberg Frisinger et al. 2021). LptD is a large protein with extracellular and periplasmic domains. It forms a stable complex with the smaller protein LptE, which resides inside the LptD β-barrel (Freinkman et al. 2011) (Fig. 1a). Due to the LptD extracellular domains, this protein is an accessible target candidate for the development of novel antibiotics. The LptDE complex is structurally conserved across Gram-negative species, including important pathogens such as *Yersinia pestis, Shigella flexneri, K. pneumoniae*, and *Salmonella typhimurium* (Botos et al. 2016). This indicates that interfering with this complex would be a good strategy for targeting multiple Gram-negative pathogens. Yet, the potential of LptD as an antibiotic target has been poorly explored. Murepavadin is one example of an antibiotic that is able to interfere with LptD in *Pseudomonas aeruginosa* (Srinivas et al. 2010, Martin-Loeches et al. 2018). Murepavadin is thought to target the LptD N-terminus, which may partially explain the selectivity as this consists of around 300 residues in *P. aeruginosa*, compared to 180 in *E. coli* (Botos et al. 2016). Polymyxins are another example of OM targeting antimicrobials, which after binding to LPS disrupt the IM and OM (Li and Velkov 2019). However, as these antibiotics are not fit for the treatment of common community-acquired infections, the need for novel antibiotics remains.

Traditional molecular methods for studying gene function are not appropriate for functional analysis of essential genes due to their constitutive requirement for cell viability. With the advent of CRISPR, new tools have been added to the microbiological toolbox. One of these is a CRISPR derivative, CRISPRi, which utilizes two point mutations in the enzyme active site leading to catalytic inactivation of the Cas9 protein, generating a so-called “dead” Cas9 (dCas9) (Qi et al. 2013). Coupled with a gene-specific single guide RNA (sgRNA), this complex causes physical blockage of the RNA polymerase, thereby silencing the expression of the target gene (Fig. 1b). This technological advancement has enabled the repurposing of the CRISPR platform from a gene-editing to a gene-silencing technology, generally referred to as gene knockdown (Larson et al. 2013). Today, CRISPRi provides a useful tool that can be harnessed to understand the function of essential genes and study the effects of gene silencing on cell viability and metabolism.

We applied CRISPRi to investigate the effects of *lptD* silencing on cell growth, morphology, and transcriptome in *E. coli*. Following efficient and titratable knockdown of the gene, measurements of gene expression showed that a high fold of gene repression could be achieved without affecting strain growth. The transcriptional response to *lptD* inhibition was investigated along with the morphological changes over time, showing that the response to knockdown is both time- and inducer concentration-dependent and that LptD depletion has profound effects on the transcriptional response with a marked upregulation in carbohydrate metabolism.

## Materials and methods

### Strains, media, and culture conditions

*Escherichia coli* BW25113 CRISPRi strains were grown in Luria-Bertani (LB) broth at 37°C, 180 rpm overnight. Kanamycin plates (30 µg ml^−1^, Gibco, UK) were used to select for the CRISPRi strains. CRISPRi knockdown was induced using IPTG (Invitrogen Life Technologies, USA) at the desired concentration. Strains and their functions are listed in Table 1.

**Table 1.** Strains used in the study.

Strain	Function
*Escherichia coli* BW25113	Parental strain ± CRISPRi system
*Escherichia coli* MFD*pir*	Helper strain during triparental mating

### Construction of mobile-CRISPRi plasmids and strain

*Streptococcus pyogenes* dCas9 was expressed from the IPTG-inducible P_LAC_promoter (#119271, Addgene). The sgRNA design was based on reported sequences (Wang et al. 2018). To make the final sgRNA selection, several sgRNAs were tested, and the one showing the greatest knockdown efficiency using growth curves was selected (Table 2). sgRNAs were annealed and cloned into the plasmid pJMP1339 using Golden Gate assembly, as previously described (Chen et al. 2017). The plasmids used to generate the CRISPRi knockdown mutant (Table 3) were cloned in the *E. coli* MFD*pir* strain. Tri-parental mating using MFD*pir* carrying either pJMP1339 or pJMP1039 (#119239, Addgene) together with *E. coli* BW25113 was performed, transferring the system to *E. coli* BW25113 for chromosomal integration downstream of *glmS* through conjugation without disrupting the gene, as previously described (Peters et al. 2019), and thus generating the final knockdown mutant.

**Table 2.** sgRNA sequences, including overhangs used for Mobile-CRISPRi, used in this study.

sgRNA	Sequence Frw (5′-3′)	Sequence Rev (5′-3′)
*lptD*	TAGTAATCATGGTGGCCAGGAGAG	AAACCTCTCCTGGCCACCATGATT
NC_1360	TAGTCCCGTATCGGCTTCCATGCA	AAACTGCATGGAAGCCGATACGGG
NC_163	TAGTAGCAGCGACCCCCTGCGAAT	AAACATTCGCAGGGGGTCGCTGCT
NC_1212	TAGTCAGAGTTATACGAATGTCTA	AAACTAGACATTCGTATAACTCTG
NC_118	TAGTAAGACTTGCTTGGCAGTCTA	AAACTAGACTGCCAAGCAAGTCTT

**Table 3.** Plasmids used to generate the CRISPRi knockdown mutant.

Plasmid	Function
pJMP1339	Contains CRISPRi system
pJMP1039	Mating plasmid

### Cell viability after CRISPRi knockdown

The effect of the cloned CRISPRi system was assessed through growth curves over 24 h using Bioscreen C (Labsystems Oy, Helsinki, Finland). Uninduced cells were grown overnight, diluted 1:100, and allowed to grow for 2 h at 37°C, 180 rpm to reach the early exponential phase. Cells were subsequently diluted down to working concentration OD_600_ = 0.01 and added to a Bioscreen Honeycomb 100-well plate containing 2x final concentration IPTG, making the final volume 200 μl. Growth curves were performed at 37°C, with the shaker set to “high” and optical density measured at OD_600_.

### Light microscopy

Cells were grown and prepared as described above. The cultures were split, IPTG was added, and the cells were grown at 37°C, 180 rpm. Samples were taken at the specified time point and stained using Nigrosin. Cells were imaged using a Zeiss Axioplan 2 microscope with an Axiocam 702 Mono and a Plan-NEOFLUAR 100x Oil Ph 3 objective lens. Images were analyzed using Fiji/ImageJ (Rueden et al. 2017, Schindelin et al. 2019).

### Electron microscopy

Cells were prepared and pre-grown as previously described. The culture was grown in the presence of 1 mM IPTG for 6 h and harvested by centrifugation. Samples were prepared as previously described (Andersen et al. 2021). Semi-thin sections (2 μm) were cut using a glass knife and ultramicrotome (Leica Ultracut, Leica Microsystems, Wetzlar, Germany) and stained with 1% toluidine blue (Millipore) and 1% Borax (LabChem). Ultra-thin sections (50–70 nm) were cut using a diamond knife (Jumdi, 2 mm) and the ultramicrotome, contrasted in 2% uranyl acetate (Polyscience) and lead citrate (Merck). The samples were examined in a Philips CM100 transmission electron microscope, and images were obtained using an Olympus Morada camera and iTEM software (Olympus).

### RNA isolation

Cells were pre-grown and prepared as previously described. Cells were harvested at specified time points post-induction by pelleting at 10 000 rpm for 5 min at 4°C, and supernatant was discarded. Cell pellets were stored at −80°C until RNA was isolated using RNeasyMini Kit (Qiagen, Sollentuna, Sweden). The quantity and quality of isolated RNA were determined by A260/280 and A260/230 ratio measurements, respectively, using a NanoDrop1000 spectrophotometer (ThermoScientific, Hvidovre, Denmark).

### RT-qPCR

Cells were grown as described above with 0 mM or 0.10–0.15 mM IPTG and harvested at time points 0-, 2-, 4-, and 5-h post-induction. DNA digestion was done using TURBO™ DNase kit (2 U/μl) (Ambion, Life Technologies, Nærum, Denmark) to purify RNA samples from contaminating DNA. Reverse transcription was used to convert RNA to cDNA using High Capacity cDNA Reverse Transcription Kit (Life Technologies, Nærum, Denmark). RT-qPCR was subsequently performed on the cDNA using FastStart Essential DNA Green Master (Roche, Hvidovre, Denmark) and a LightCycler 96 (Roche, Hvidovre, Denmark). Primers used to detect *lptD* were lptD-F: TGGTATCGCCCTGTACCAGA and lptD-R: TGATTGCCACCGCCCTTTAT, and for the control gene *gapA*, gapA-F: ACTGACTGGTATGGCGTTCC and gapA-R: GTTGCAGCTTTTTCCAGACG. Change in gene expression was calculated using the 2^−ΔΔCT^ method.

### RNA-seq

Cells were grown as previously described using seven different conditions: 0.1 mM IPTG, 1 mM IPTG, 0.04 μg/ml CST, 0.06 μg/ml CST, 0.1 mM IPTG + 0.04 μg/ml CST, 0.1 mM IPTG + 0.06 μg/ml CST, or without IPTG (control). Each of these seven conditions was tested in four replicates, leading to a total of 28 samples analyzed by RNA Seq. Cells were harvested at 4.5 h post-induction by centrifugation at 4°C, 4000 rpm. Pellets were snap-frozen in liquid nitrogen and stored at −80°C. RNA was isolated as described above. From each sample, 400 ng of total RNA was used to prepare the library for RNA-Seq following RNAtag-Seq protocol (Shishkin et al. 2015), which was sequenced on Illumina NextSeq500. The bcl2fastq software (v2.19, Illumina BaseSpace) was used to convert raw data to fastq files, and RNA-seq data were processed using an in-house analysis pipeline as previously described (Love et al. 2014, Zhu et al. 2020). Finally, gene expression dataset was uploaded to the BioCyc Omics Dashboard (Paley et al. 2017) for enrichment analysis.

## Results

### Characterizing the effects of *lptD* gene silencing on *E. coli* growth

**Figure 2.**
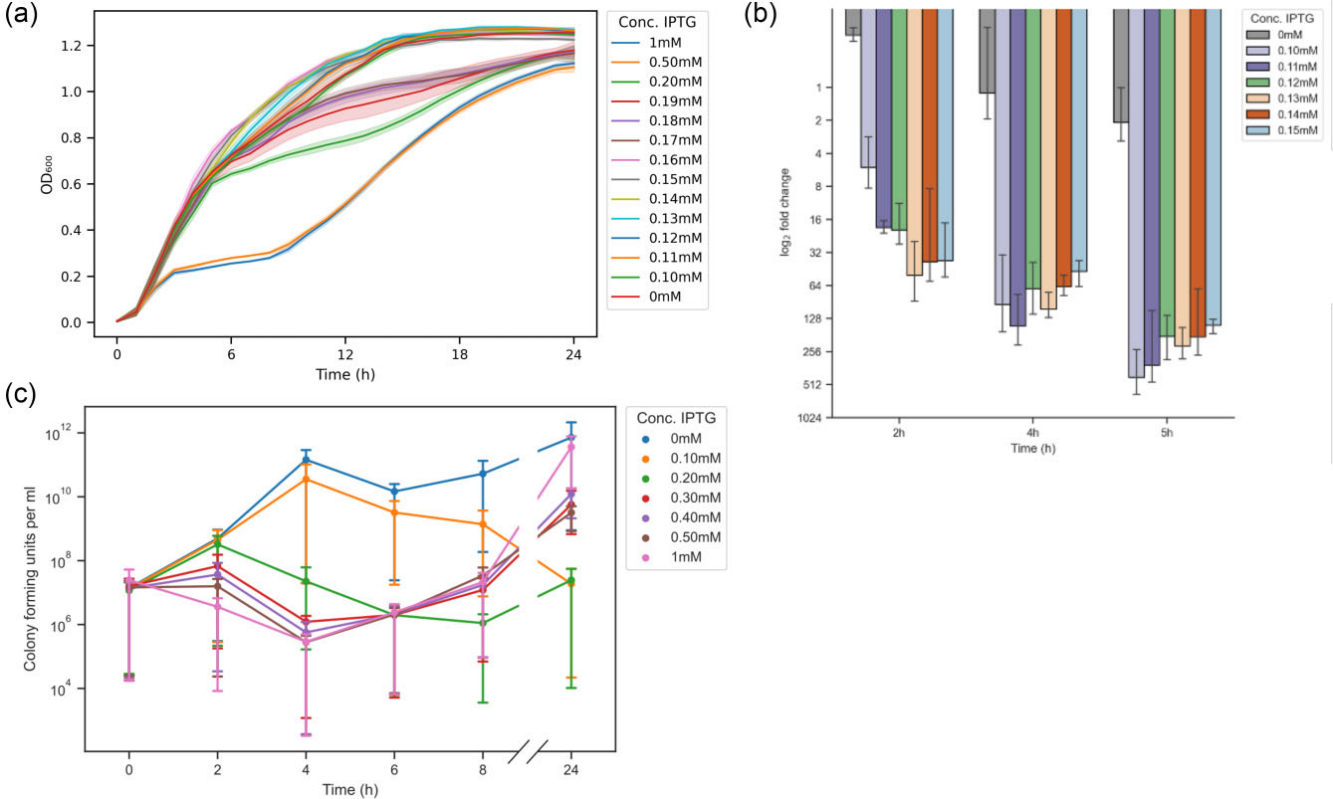
**(a)** Growth curves of *E. coli lptD* knockdown mutant grown in LB broth with different concentrations of IPTG. Data represent the average of three biological replicates. **(b)** Expression of *lptD* over time quantified by RT-qPCR using increasing concentrations of inducer relative to the housekeeping gene *gapA*. Data represent the average of three biological replicates. **(c)** Colony-forming units at various concentrations of IPTG. *Y*-axis shows log-transformation of cell counts. Error bars are SDs of three technical replicates.

To investigate the effects of *lptD* knockdown, the CRISPRi construct was grown under a range of IPTG inducer concentrations (0.1–1 mM). Severe growth inhibition was observed for the first 9 h under high concentration of IPTG (1 mM), followed by exponential growth reaching saturation after ∼18 h (Fig. 2a). Growth under low concentrations of IPTG (0.10–0.20 mM) exhibited a dose-dependent response (Fig. 2a). At concentrations ≤0.17 mM growth was similar to that of the uninduced control, indicating a threshold for observable knockdown (Fig. 2a).

Overexpression of the CRISPRi system has previously been shown to result in toxicity in *E. coli*, leading to a perturbed growth pattern (Cho et al. 2018, Cui et al. 2018). To rule out any non-specific effect due to the expression of the recombinant system, we developed control strains containing a functioning CRISPRi system but with non-targeting sgRNAs (NC_1360, NC_1212, NC_163, and NC_118). None of the strains exhibited growth inhibition even upon strong induction of the system (Supplementary Fig. S1). This indicates that the growth reduction of *E. coli lptD*-CRISPRi strain observed in the previous experiments was due to silencing of *lptD* expression.

To investigate the degree of CRISPRi-mediated silencing on *lptD* transcription, we measured the reduction in gene expression upon subinhibitory induction of the CRISPRi system (Fig. 2b). Low levels of inducer were sufficient to generate a high-fold knockdown, which is in accordance with previous findings (Peters et al. 2016). Although *lptD* knockdown was inducer concentration-dependent at the earlier time points (2 h post-induction), the highest repression (∼440-fold reduction) was observed at 5 h post-induction using 0.10 mM IPTG, compared to the ∼128-fold reduction achieved with 0.15 mM at the same time point. The impact of concentrations ≤0.13 mM on cell growth became visible only after 5 h (Supplementary Fig. S2). Together, these results show that efficient gene silencing can be achieved with low induction of the CRISPRi system, although there is a lag between gene depletion and any effect on growth as measured by turbidimetry.

To further investigate the impact of LptD depletion on cell viability, we carried out viable cell counts (Fig. 2c) upon induction of the CRISPRi system. High levels of inducer (0.30–1 mM) led to a decrease in viable cells up to 4 h, when the cells started to grow back to the same levels as the control (∼10^12^ CFU/ml) (Fig. 2c). Whole-genome sequencing of strains isolated following growth in 1 mM IPTG showed inactivating mutations in the CRISPRi system, thus creating suppressor mutants allowing the cells to escape the knockdown effect (Supplementary Fig. S3). Cultures exposed to the lowest level of inducer (0.10 mM) exhibited a completely different pattern: growth was identical to that of the control up until 4 h post-induction, after which a clear decline in the number of viable cells was observed (Fig. 2c). No recovery from this downward trend was observed at the following time points, resulting in very low numbers (∼10^7^ CFU/ml) of viable cells after 24 h. Conversely, cells exposed to an intermediate IPTG concentration (0.20 mM) showed a complex behavior of initial duplications, followed by a decline in numbers after 2 h and a subsequent increase after 8 h and until the experimental end-point (24 h). These results show that high levels of induction lead to suppressor mutants that inactivate the CRISPRi system and grow back to wild-type cell numbers. On the contrary, low levels of induction appear not to lead to suppressor mutants under the experimental conditions tested.

### LptD depletion disrupts *E. coli* morphology

**Figure 3.**
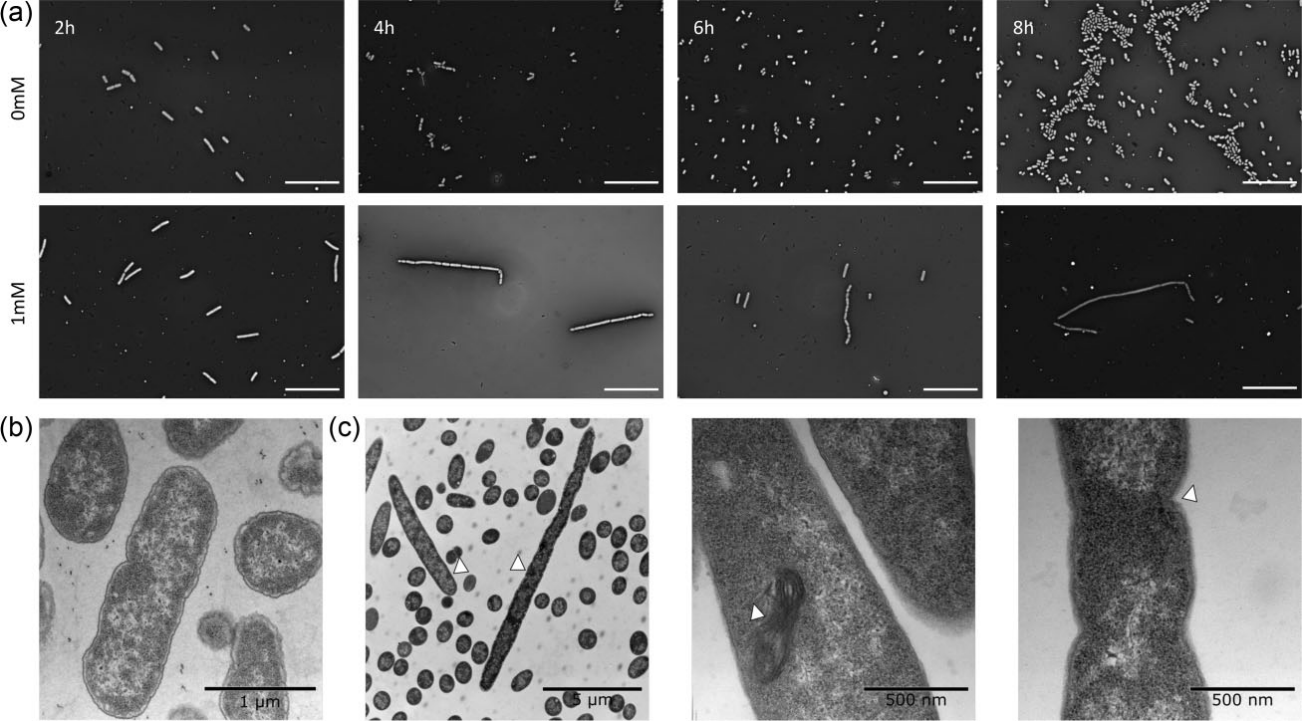
Knockdown of *lptD* using CRISPRi leads to morphological changes over time. **(a)** Micrographs of the induced (1 mM) or uninduced (0 mM) CRISPRi strain sampled at 2, 4, 6, and 8 h. Scale bars: 200 µm. **(b)** TEM of uninduced control and **(c)** induced (1 mM) cells grown for 6 h, which show elongation and deterioration in cell shape.

The OM is integral for maintaining cell shape in Gram-negative bacteria, and an imbalance of its composition can have detrimental effects on cell morphology. To investigate the effect of LptD depletion on cell morphology, we employed light microscopy and transmission electron microscopy (TEM) to visualize uninduced and fully induced (1 mM) cells over time. Following growth 2 h post-induction, non-homogenous morphologies were observed with a mix of elongated, smooth cells and multi-septated, filamentous cells (Fig. 3a). This change in morphology continued up to 8 h, with additional deterioration of cell shape. This phenomenon was also observed under mild depletion, starting from 0.20 mM, and the effect increased with inducer concentration, although cells appeared to return to normal morphology at 24 h (Supplementary Fig. S4), most likely due to CRISPRi inactivation. These morphological changes appeared to follow a different timeline than the growth experiments. For example, following 0.20 mM induction, the altered morphologies were observed at the earlier time points, but cell morphology was largely restored after 24 h growth (Supplementary Fig S4). In contrast, the growth experiments showed a reduced population size after 24 h but with an increased growth rate between 8 and 24 h (Fig 2c), indicating a lag between the development of suppressor mutations inactivating the CRISPRi system and restoral of the cell population density.

TEM showed no effect on the uninduced control (Fig. 3b), whereas the induced strain exhibited elongated, filamentous morphologies (Fig. 3c). Cells appeared visually denser and with a reduced periplasm, in line with intracellular accumulation of LPS (Sperandeo et al. 2008). This shows that LptD depletion causes drastic changes in cell morphology.

### The transcriptional response to *lptD* knockdown centers around carbohydrate metabolism

To reveal any genome-wide response to *lptD* knockdown, we profiled and compared the transcriptomes of the induced and uninduced *lptD*-CRISPRi strain at 4.5 h post-induction. While only 22 genes were significantly (log_2_FC > 1 and *padj* < 0.05) regulated in response to the presence of 0.10 mM IPTG, the expression of 752 genes changed significantly due to the knockdown of *lptD* using 1 mM IPTG (Supplementary Table 1, Supplementary Fig. S5a, b). Only five genes, in addition to *lptD*, were found to be shared between the two conditions: *pdxA* (4-hydroxy-L-threonine phosphate dehydrogenase), *prpR* (propionate catabolism operon regulatory protein), *yahO* (uncharacterized protein), and two IS elements. This indicates that the cell experiences mild and severe levels of LptD depletion differently. Additionally, *pdxA* belongs to the same operon as *lptD* but is located one gene downstream of the CRISPRi-targeted gene and can be expressed using *lptD*-independent promoters (Tramonti et al. 2021), making it challenging to interpret whether the observed change is due to polar effects or a true response to LptD depletion.

**Figure 4.**
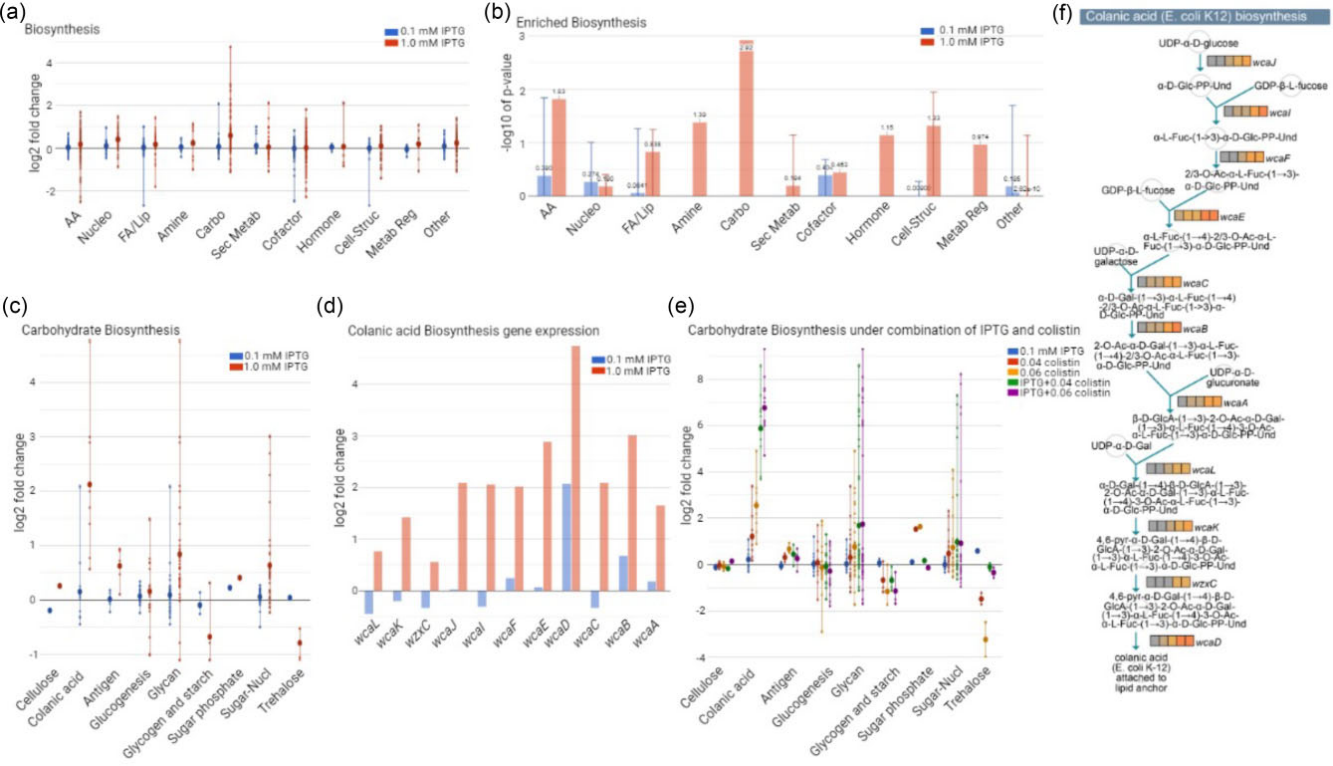
Expression change of biosynthesis and colanic acid-related genes. **(a)** Average expression changes of individual subsystems involved in biosynthesis processes are presented in large dots. Small dots represent the fold-change value of individual genes of the subsystem. **(b)** Enrichment scores of individual biosynthesis processes are presented as −log_10_ of the *P*-value of enrichment. **(c)** Average expression changes of the various carbohydrate biosynthesis pathways. **(d)** Fold change of expression of the different genes found in the colanic acid biosynthesis pathway. **(e)** Average expression changes of the various carbohydrate biosynthesis pathways under LptD depletion and/or colistin exposure. **(f)** The colanic acid biosynthesis pathway in *E. coli*.

Individual replicates exposed to IPTG clustered together and separated well from the 0 mM IPTG control (Supplementary Fig. S6a, b), confirming the accuracy of performance of the experiment, processing, and analysis of RNA-Seq samples. To identify modulated metabolic and cellular processes, the gene expression dataset was further explored using the Omics Dashboard of BioCyc (Paley et al. 2017). Multiple anabolic and catabolic processes were found to be up- or down-regulated as a response to LptD depletion. Carbohydrate metabolism-related processes were the most significantly upregulated (Fig. 4a and b). Remarkably, GDP-sugar metabolism was highly upregulated in the presence of 1 mM IPTG, and most of the genes of this subsystem were overexpressed (Supplementary Fig. S7a). In particular, the GDP-L-fucose biosynthesis pathway was upregulated, especially the genes *fcl* and *gmd* (Supplementary Fig. S7b). Moreover, *lptD* knockdown was found to lead to the downregulation of *lpxK*, which is involved in the synthesis of tetraacyldisaccharide lipid (IV)A by phosphorylating the precursor disaccharide-1-phosphatedisaccharide (Supplementary Table 1). The lipid A core is the foundation of the LPS molecule, and the reduction of lipid (IV)A levels likely slows down the rate of the entire LPS biosynthesis process.

### Targeting LPS transport upregulates colanic acid biosynthesis

The most significantly upregulated carbohydrate biosynthesis pathway was that for colanic acid biosynthesis (Fig. 4c). All genes of this polysaccharide biosynthesis pathway were overexpressed in the presence of 1 mM IPTG but not for 0.10 mM IPTG (Fig. 4d). Recently, it has been proposed that colistin binds to LPS at both the IM and the OM, ultimately causing cell lysis (Sabnis et al. 2021). We hypothesized that knockdown of *lptD* increases LPS levels in the IM and potentiates colistin. Indeed, 0.10 mM IPTG was able to potentiate colistin, where the combination exposure led to a stronger growth reduction compared to colistin alone (Supplementary Fig. S8).

To test whether the colistin-LptD depletion interaction is linked to the upregulation of the colanic acid biosynthesis pathway, we profiled the transcriptome of the *lptD*-CRISPRi strain grown in the presence of IPTG and colistin individually and in combination. To capture the reproducibility and sensitivity of the transcriptional response, two sublethal colistin concentrations (0.06 and 0.04 µg/ml) were selected, which exhibited ∼50% and ∼40% growth inhibition, respectively, compared to the unexposed control (Supplementary Fig. S8). Remarkably, 0.10 mM IPTG highly upregulated the colanic acid biosynthesis in combination with colistin regardless of the drug concentration (Fig. 4e). The entire pathway (Fig. 4f) was strongly activated by the combination. Conversely, this low concentration of IPTG was not sufficient to modulate the exopolysaccharide biosynthesis pathway, and colistin alone only moderately upregulated this pathway (Fig. 4e). It has previously been reported that phosphorylation of Ugd (UDP-glucose dehydrogenase) by tyrosine-kinase Wzc induces the production of colanic acid, and phosphorylation of Ugd by Etk induces capsule synthesis (Obadia et al. 2007, Lacour et al. 2008). Ugd, the Wzc kinase, and the Wzb phosphatase were all overexpressed in the presence of colistin and highly upregulated when cells were grown in the presence of IPTG and colistin combination, while Etk was downregulated (Supplementary Table 2).

### Reduced LPS transfer impairs motility

**Figure 5.**
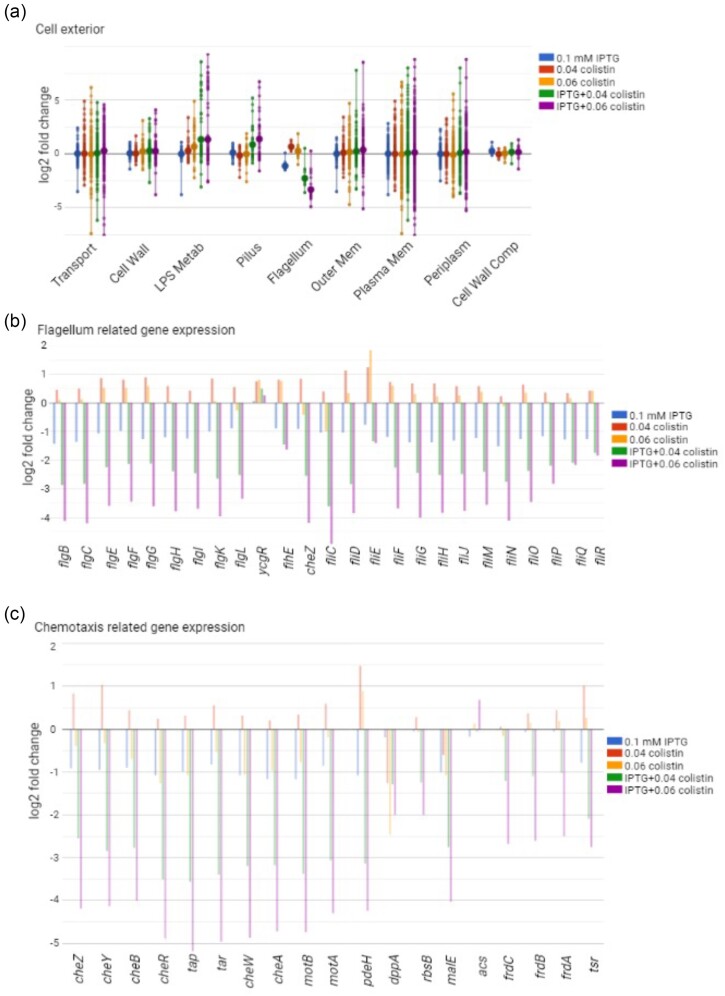
Expression changes as a result of LptD depletion and/or colistin exposure. **(a)** Expression changes in the cell exterior pathways. The larger dots represent the average expression changes of the individual subsystems, and the smaller dots represent individual genes within the subsystem. **(b)** Expression changes in the flagellum-related genes. **(c)** Expression changes in the chemotaxis-related genes.

Among the processes related to the cell exterior, the LPS metabolism subsystems were upregulated when cells were grown in the presence of IPTG and colistin together (Fig. 5a). Both flagellar and chemotaxis proteins, which determine the locomotion of the cell, were downregulated in the presence of 0.10 mM IPTG but upregulated in the presence of colistin (Fig. 5a). The combination of IPTG and 0.04 or 0.06 µg/ml colistin strongly downregulated almost all the genes of these two processes (Fig. 5b and c). c-di-GMP plays a central role in the regulation of flagella biogenesis by modulating the function of regulator YcgR (Guttenplan and Kearns 2013), where the c-di-GMP binding form of YcgR inhibits the production of flagella. The intracellular levels of c-di-GMP are in turn regulated by the PdeH (c-di-GMP phosphodiesterase). While exposure to colistin alone increased the expression of *pdeH*, the combination of IPTG and colistin strongly downregulated the *pdeH* levels (Fig. 5c). This suggests that the combination of colistin and *lptD* knockdown increases levels of c-di-GMP, which in turn inhibits the biogenesis of flagella by binding to and inactivating YcgR.

## Discussion

The present study describes the response of *E. coli* to LptD depletion using a CRISPRi system that allows modulation of *lptD* gene expression. The CRISPRi construct exhibited severe growth inhibition upon strong induction of the CRISPRi system, whereas a sublethal and tuneable response was observed at lower inducer concentrations.

We were able to show an efficient knockdown at sub-inhibitory inducer concentrations, suggesting either a protein surplus or slow protein turnover, allowing survival during LptD depletion. LptD has previously been reported to be a low-abundance protein with an estimated 60 molecules per cell (Link et al. 1997), suggesting that the latter scenario may be the case. In support of this hypothesis, it has been previously shown that LptD depletion is slow and can take four to five generations before the associated phenotypic effects are observable (Braun and Silhavy 2002). Our results indicate that this lag time depends on the level of depletion and that cells can survive under repressive conditions for up to 4 h before a change in growth can be observed. The LptDE complex forms an extremely stable macromolecule in the OM (Chng et al. 2010). Thus, the growth inhibitory effects under mild inhibition may be related to LptD dilution through cell division, rather than protein degradation leading to an LptD shortage.

LptD depletion was found to be associated with morphological changes. Previous studies on LptD depletion have found this to be associated with filamentation, loss of membrane integrity, and cell lysis (Braun and Silhavy 2002), or membranous bodies protruding into the periplasm (Sperandeo et al. 2008). Abnormal periplasmic membrane structures were not observed in the present study. Instead, cells were found to become highly elongated, displaying either a smooth or filamentous morphology. This discrepancy in phenotype observed between different studies may be related to the LptD depletion levels, which in turn may be affected by methodology making direct comparisons difficult. Regardless, it is clear that the cells were heavily impacted as a result of LptD depletion, as the controls containing function CRISPRi-system with non-targeting sgRNAs induced at the same level did not exhibit an altered morphology.

The transcriptional response to LptD depletion appeared to mainly revolve around carbohydrate metabolism. Although various biosynthesis and degradation processes were found to be up-or down-regulated, it is interesting to note that the most prevalent responses were related to an upregulation of carbohydrate metabolism. Additionally, LptD depletion in combination with colistin exposure was found to reduce the expression of several genes involved in motility. Motility is essential for bacterial pathogens in certain growth phases and is intimately linked with virulence (Josenhans and Suerbaum 2002). Increased expression of motility-related genes has also been associated with strains capable of causing persistent bovine mastitis when compared to strains causing transient infection (Lippolis et al. 2018). The reduced expression of motility genes may indicate that the cells are less likely to establish persistent infection, and thus targeting LptD may be an attractive way to control infection.

Expression of all genes involved in the colanic acid biosynthesis pathway increased strongly in the presence of IPTG and colistin in combination, suggesting that LptD depletion potentiates colistin and cells increase production of colanic acid exopolysaccharide in response to the envelope damage caused by the antibiotic. Conditional LptD depletion has previously been shown to lead to an accumulation of LPS decorated with colanic acid in the IM in *E. coli* (Sperandeo et al. 2008). Furthermore, biosynthesis of colanic acid in *E. coli* has been shown to be dependent on LPS structure (Ren et al. 2016, Wang et al. 2020). Studies of the proteomic response to LptC depletion in *E. coli* found this to be linked to an upregulation of proteins involved in envelope biogenesis, including three proteins involved colanic acid biosynthesis (Martorana et al. 2014). Using transcriptomic analysis of *lptD* knockdown, we successfully showed an upregulation of all 11 genes of the colanic acid biosynthesis pathway. Our results indicate that the abundance of LPS in the OM and the condition of the cell envelope ultimately modulate colanic acid production. The extreme upregulation of colanic acid biosynthesis observed only under the combination treatment indicates a synergistic action between colistin and *lptD* knockdown. Colanic acid has been reported to play an important role in biofilm formation (Danese et al. 2000), which is tightly coupled with motility (Guttenplan and Kearns 2013). Transiting from planktonic to biofilm growth might be a strategy to overcome the stress of envelope damage. Here, we show that the overexpression of colanic acid exopolysaccharide is a potential strategy of *E. coli* to impede the potency of an envelope-damaging agent like colistin or to maintain envelope integrity. Our results also indicate that targeting of LPS transport induces a complex transcriptional response, revealing an intricate connection between colanic acid biosynthesis, motility, and biofilm growth. It would be interesting to follow up on these results by investigating the effects of *lptD* depletion on the Rcs system, which is important for sensing OM damage and is a positive regulator of colanic acid biosynthesis (Meng et al. 2021).

Protein depletion using CRISPRi is associated with drawbacks, including off-target and the bad-seed effect, which are both linked to the sgRNA design. To avoid any off-target effects, the sgRNAs used in the present study were based on previously published designs following strict rules to avoid off-target effects (Wang et al. 2018). Previous findings have shown that certain “seed sequences” within sgRNAs are associated with unexpected toxicity, which is unrelated to the gene expression modification, known as the “bad-seed” effect (Cui et al. 2018). To avoid such unwanted and non-specific toxicity, the selected sgRNAs did not contain seed sequences associated with the bad-seed effect. Another drawback of CRISPRi mediated expression regulation is potential polar effects, which arise when the CRISPRi system prevents transcriptional readthrough of genes downstream of the intended target. In *E. coli, lptD* is part of a complex, multifunctional operon comprising the genes *lptD, surA, pdxA, rmsA*, and *apaGH*, containing at least eight different promoters capable of giving rise to a range of different transcripts (Tramonti et al. 2021). Any such unintentional regulation of these genes may result in additional effects on the cell that could be misinterpreted as being due to LptD depletion, especially *surA*, which assists in the folding of certain OM proteins (Lazar and Kolter 1996). However, none of the genes present in this operon exhibited significant changes in gene expression apart from *pdxA*, indicating little or no polar effect on the remaining genes.

LPS is essential in the majority of Gram-negative bacteria, where the tight packaging and negative charge of the LPS molecules act to exclude extracellular toxic stressors, conferring intrinsic resistance toward many noxious substances (Bertani and Ruiz 2018). Disruption of the OM has been shown to overcome intrinsic, acquired, and spontaneous antibiotic resistance by allowing intracellular access or by overcoming many antibiotic inactivation determinants while reducing the rate of spontaneous resistance development (MacNair and Brown 2020). Furthermore, LPS plays an important role in Gram-negative virulence and pathogenicity. In a recent study, a non-pathogenic strain of *E. coli* exhibiting 500-fold higher virulence than the parental strain was isolated from a silkworm infection. This was partially attributed to LptD and LptE mutations, leading to increased secretion of LPS containing OM vesicles and endowing the strain with heightened resistance toward various antibiotics, antimicrobial peptides, and host complement (Kaito et al. 2020). Reducing the surface LPS by inhibiting the Lpt pathway may therefore aid in reducing the virulence of a pathogen. Thus, targeting LptD may be a good strategy for reducing membrane stability and increasing permeability, thereby potentiating otherwise inactive antibiotics while additionally decreasing virulence, which would aid in the clearance of infection.

Despite its merits, the study presented here has limitations, and some findings could be verified by further research. It would be interesting to experimentally measure whether CRISPRi-mediated LptD depletion causes intracellular accumulation of LPS. In addition, RT-qPCR could be used to confirm the transcriptional changes in genes critical to the discussion (*surA, pdxA, rmsA*, and *apaGH*).

## Supplementary Material

xtad013_Supplemental_Files

## Data Availability

Raw RNA-seq datasets are available at the Sequence Read Archive (BioProject accession number ID PRJNA810754).
