# Supplementary material for: LptD depletion disrupts morphological homeostasis and upregulates carbohydrate metabolism in Escherichia coli
Source: FEMS Microbes. 2023 Aug 10;4:xtad013. doi: 10.1093/femsmc/xtad013 (PMC10495129; doi:10.1093/femsmc/xtad013)
Supplement: xtad013_Supplemental_Files [file xtad013_supplemental_files.zip › Supplementary_figures_final.docx]

**Figure S1.** Growth curves of control strains containing a functioning CRISPRi system but with sgRNAs lacking targeting activity (negative controls), under different concentrations of inducer.

**Figure S2.** Growth curves of cultures used for RT-qPCR analyses.

**Figure S3.** Growth curves of suppressor mutants grown under different concentrations of inducer.

**Figure S4.** Micrographs of CRISPRi-*lptD* cells grown under different concentrations of inducer at different time points. Scale bars: 200 μm.

**Figure S5.** Volcano plot of RNAseq data of cells grown in the presence of 0.1 (A), and 1 (B) mM IPTG.

**Figure S6.** Clusters of replicates of RNAseq samples of cells grown in the presence of 0.1 (A), and 1 (B) mM IPTG.

**Figure S7.** (A) Subpathways up- and down-regulated in the sugar-nucleotide biosynthesis pathway as a result of exposure to 0.10 or 1mM IPTG. The smaller dots represent individual data points, and the larger dots represent the average. (B) Genes up- and down-regulated in the GDP-sugar biosynthesis gene expression pathway.


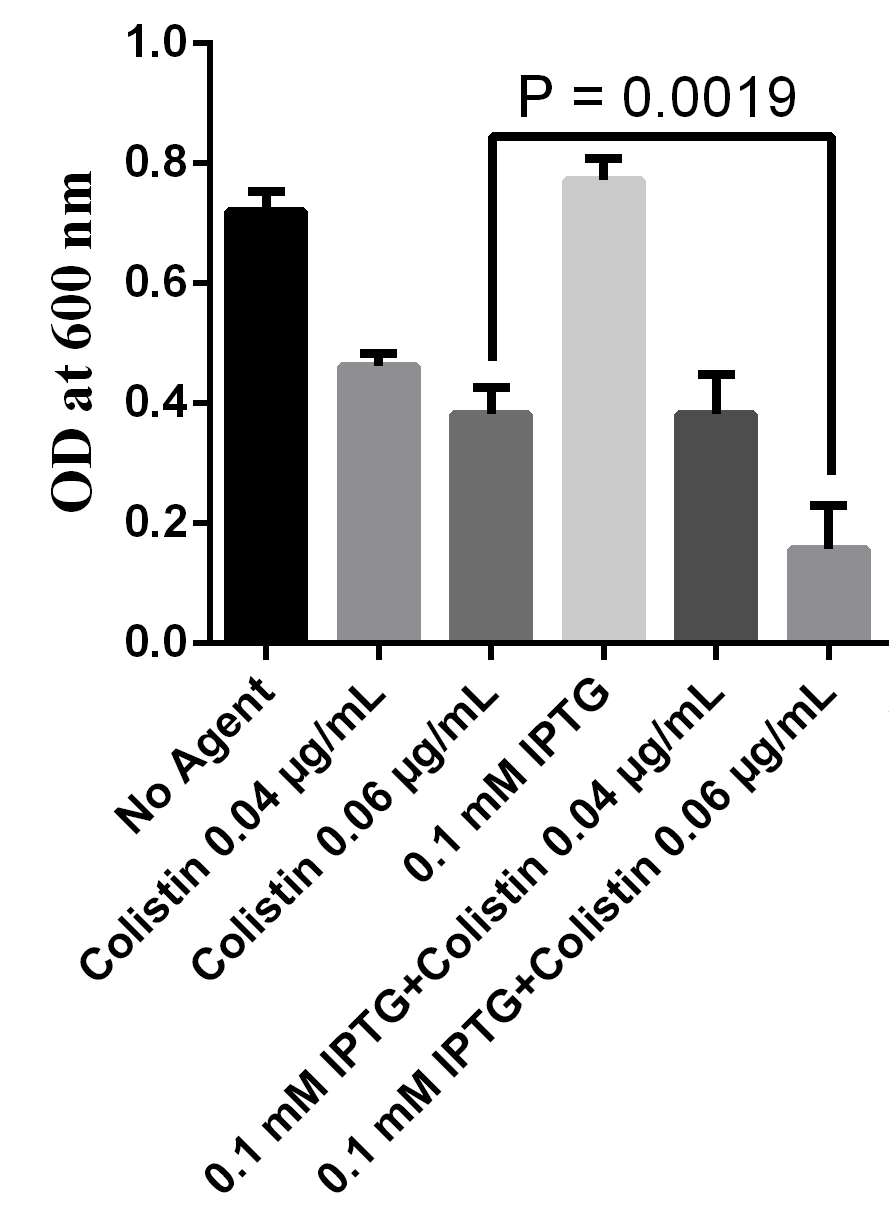


**Figure S8.** Growth of the CRISPRi-*lptD* strain after 4.5 h of growth under different conditions with or without IPTG (0.1mM) and colistin (0.04 or 0.06 μg/mL) compared to the uninduced and unchallenged control.
