# Supplementary material for: LptD depletion disrupts morphological homeostasis and upregulates carbohydrate metabolism in Escherichia coli
Source: FEMS Microbes. 2023 Aug 10;4:xtad013. doi: 10.1093/femsmc/xtad013 (PMC10495129; doi:10.1093/femsmc/xtad013)
Supplement: xtad013_Supplemental_Files [file xtad013_supplemental_files.zip › Supplementary_Data_-_FEMSMC-2022-028_reviews_and_author_response.docx]

**Reviews of manuscript FEMSMC-2022-028**

**Reviewer 1**

**Recommendation: Minor Revision**

The manuscript by Svanberg Frisinger and co-workers reports on the construction and characterization of a CRISPRi strain in E. coli that allows the tuneable expression of the essential lptD gene. In this work, after confirming the appearance of envelope biogenesis-related phenotypes in the strain under induction of the repression system, the authors analyse the changes in the transcription profile upon LptD depletion. From this analysis the authors identify the upregulation of colanic acid biosynthesis genes as the strongest effect of LptD depletion, that they interpret as a mean of the cells to fortify the damaged envelope. Finally, from the analysis of the combination of LptD depletion and colistin treatment the authors observe an even stronger increase in colanic acid biosynthesis gene expression associated to the downregulation of flagella synthesis and chemotaxis genes, indicative of a switch from planktonic to biofilm growth mode. This latter finding has important implications in bacterial virulence.

The overall work is interesting for the readership of the journal, and the topic is relevant. The construction of the LptD depletion strain is a valuable tool to assess the synergy between drug treatments and outer membrane damage. However, the analysis of the transcriptomic data is sometimes too much speculative and lacks appropriate validations to support the main conclusions of the study.

Following I report some comments/suggestions I believe that could help making the manuscript clearer and strengthening the conclusions of the work:

1) The first part of the work is focused on the adaptation of the CRISPRi system to allow the knockdown of lptD, with the aim of identifying the suitable concentration of the inducer that leads to gene repression avoiding the appearance of mutations that inactivate the repression system. The analysis is thorough, but I think that it would be useful to accompany the transcriptional analysis with the analysis of the steady-state level of the LptD protein by western blot under different CRISPRi induction conditions. This would help understanding the degree of protein depletion obtained.

2) After the initial characterization, the authors test whether the knockdown strain displays the known phenotypes associated to LptD depletion and therefore assess cell viability and cell morphology using light microscopy and TEM. The morphological analysis using full (1 mM) and mild (0,2 mM) induction conditions at different time points shows the appearance of a filamentous phenotype of the cells that turns to normal after 24 h and the authors explain this phenomenon with the appearance of suppressor mutations. However, in Fig 2C the viability of the cells treated with 0.2 mM IPTG seems not to be restored at 24 h post induction. Could the authors please clarify on this?

3) At line 172, the authors suggest that the reduction in the size of the periplasm, visible in Fig. 3C, is indicative of LPS accumulation. I suggest the authors indicate the reference for this statement. The accumulation of LPS in the inner membrane is also mentioned as responsible of the downregulation of lpxK gene expression at line 206, however LPS accumulation in the inner membrane under these conditions is not confirmed experimentally. I think that the authors should demonstrate the LPS accumulation in the inner membrane by western blot using anti LPS antibody on inner and outer membrane fractions.

4) In the paragraph starting from line 181, the authors mention 3 genes (pdxA, prpR and yahO) that are differentially regulated compared to the uninduced conditions under both the induction conditions analysed (0.1 and 1 mM IPTG), but no comments are made on this, and the relevance of this data is not clear. Could the authors please comment on this?

5) The synergy between lptD knockdown and colistin treatment is interesting and the RNAseq analysis suggests that the combination of the two conditions is associated to the upregulation of colanic acid biosynthesis genes. The authors suggest that this might be a strategy used by the cell to fortify the cell envelope. Also in this case, the authors should strengthen their hypothesis by quantifying the level of colanic acid produced under the conditions tested and possibly by evaluating the sensitivity to colistin of a mutant deleted for wcaJ (that is therefore not able to produce colonic acid) under lptD knockdown.

Minor points:

Figure 1A: The transmembrane helix of LptC should be inserted between LptF and LptG subunits.

Figure 2A: the authors state that the growth profile shows a dose-dependent response from 0,15 mM IPTG but I think that the effect is dose-dependent only at concentration ≥ 0.17 mM

Figure 3C: It would be useful to indicate in the low magnification image where the markers of cell deterioration that are zoomed in in the panels on the right are located

Line 207: lipid IV A derives from LpxK-mediated phosphorylation of the precursor disaccharide-1-phosphatedisaccharide, please correct.

Figure S8 should show the synergy between colistin treatment and LptD depletion, however the graph is not clear to me, and it seems that a panel is missing.

**Reviewer 2**

**Recommendation: Major Revision**

The authors examined the effect of the lptD knockdown by using CRISPRi. The experimental design is good, but the presentation of figures are too small to understand. I cannot see whether there is a difference or not in Figures 4 and 5. Also, statistical analysis should be performed to support the author's conclusion.

Main concerns

1) Fig S1, explanations for NC_1360, NC_1212, NC163, and NC_118 are needed.

2) Fig. 2, growth curves were probably obtained by measuring OD600 in microplate reader. The amount of broth in the well, temperature and shaking conditions, and microplate reader product name should be described.

3) Lines 395-401, oligonucleotide primer sequences should be presented. What gene was used for internal control? The internal control gene also should be described in the legend of Figure 2B.

4) Figures 4 and 5, the size is too small to understand the data. I cannot see the labels for the axis. In addition, is statistical analysis performed? Statistical method and the values should be presented in the legends or in the figures.

5) Figure S5, I cannot see the labels for the axis.

6)Figure S7A, what is the closed circles and the small dots? These should be explained in the legends.

7) Figure S7B, What gene was used for internal control? Was Statistical analysis performed?

**Reviewer 3**

**Recommendation: Reject**

This article by Frisinger et al. reports effects of the lptD depletion in E. coli by the CRISPRi method, including defects in cell morphology and cell growth, as well as expression pattern change of genes in carbohydrate metabolism. The authors also introduced colistin treatment to the lptD-depletion strain and identified up- or down-regulation of motility, chemotaxis, and virulence genes. Although the CRISPRi method appears simple and fast, there are some concerns needing to be clarified before interpreting the results.

1. Modification of the title “LptD depletion disrupts morphological homeostasis and rewires carbohydrate metabolism of Escherichia coli” is recommended, since this work does not directly measure the carbohydrate metabolism. Instead, gene expression is investigated.

2. Since the morphological defect was documented in Braun & Silhavy (2002), the author please discuss any difference in morphology between the previous and current studies using different methods.

3. The essential details of the CRISPRi experiments are missing.

What is the sequence of sgRNA? The cited reference of Wang et al. 2018 (line 355-6) reported 15 sgRNAs of lptD (Supplementary Data 3 and 4) and the experiment was performed with pooled sgRNAs. Which one is used in this study? How did the author select the particular sgRNA?

How were the RT-qPCR data quantified?

How was the reported fold change of gene expression defined and calculated?

What is the reference gene used in quantification?

4. The authors please address how do the drawbacks of the CRISPRi method impact this study, which include off-target, bad-seed, and polar effects.

The increasing IPTG concentration to change the expression level of dCas9 is to examine the bad-seed effect. Judging from Figure 2C and S4, IPTG at 0.2 mM could be more than sufficient to balance cell viability and morphological defect.

The off-target effect of the sgRNA used in the study needs to be discussed in detail.

There are also adjacent genes up- and downstream to lptD. Whether the polar effect occurs along with the lptD depletion needs to be examined in order to clarify whether or not any polar effect influences the observations.

5. Because of the possible bad-seed effect with dCas9 at high concentration, those experiments using 1 mM IPTG induction may not be relevant to lptD depletion.

Line 186-187: It is questionable about the relevance of the genes identified using 1 mM IPTG. Same for Figure 3C: TEM data

6. The statement for the colistin treatment on top of the lptD depletion is arguable. Since the complex is disrupted in the lptD-depletion strain, the effects seen after colistin treatment do not go through the lpt protein complex. It is more likely that the lptD depletion reduces OM integrity and the colistin treatment resulting in cell surface damage is an “additive”, instead of “synergistic”, effect. Both lptD depletion and colistin treatment can trigger stress responses in bacterial cells, but likely through different mechanisms.

7. Following point 3, the up- or down-regulated genes associating with addition of colistin in the lptD-depletion strain relates to colistin, but not necessarily related to the lptD depletion.

8. The sgRNA is integrated in the chromosome down stream of glmS (line 359) that encodes an enzyme catalyzing the first reaction in the UDP-GlcNAc synthesis pathway. UDP-GlcNAc is the precursor molecule of LPS and PG. Since Figure 4CE and S7A show changes in nucleotide sugar synthesis, the authors please comment on whether integration of the sgRNA plasmid at this chromosomal location influence glmS expression or not.

Other comments

9. Need to provide strain, plasmid, and primer tables.

10. Since LptD is a low-abundance protein (line 276-277, 60 molecules per cell), the wording of "high-fold depletion" is confusing.

11. Labels are too small in most figures.

12. “suppressor” mutant instead of “escape” mutant (line 137, 170, Figure S3)

Line 59: periplasmic

Line 70: terminus

Line 183: Is the level of leaky expression determined under the un-induced condition?

Line 189: pdxA is one of the downstream genes to lptD. Address polar effect or not.

Line 230: How does the growth reduction affect the observation?

Line 274: high-fold knockdown?

Line 275: “slow” protein turnover

Line 277: 60 molecules per cell: Is it possible to estimate a number from the RT-qPCR data?

Line 293: What is the non-coding control? With or without sgRNA? Or with or without induction of dCas9?

Materials and Methods

Lines 356-361: contain redundant information.

Line 364-: Alternatively, the cell viability can be easily tested by spotting on agar plates.

Line 395: Details of the RT-qPCR experiment are necessary.

Figure 1: (1) all symbols indicating different components need to be labeled and explained, including the outer and inner membranes. (2) (B) is a generic illustration of the CRISPRi system and does not provide specific information to lptD knock-down in this work. This figure needs to include the sequence of sgRNA, its priming site, and the location of where it is integrated on chromosome, etc.

Figure 2: need experimental details to validate 2B. Correct label of the y axis.

Figure 3: (1) The scale bar size in micrographs is not correct. (2) Presenting micrographs in (B) and (C) in the same scale is recommended. (3) In (C), use arrows or symbols to indicate features that correspond to the morphological defects or LPS accumulation, etc.

Figure 4: Too small to see.

Figure 5. Please comment on whether the growth defect considered in the experimental design or in the analysis. Too small to see.

Figure S4: missing size of scale bar

Figure S5: too small to see

Figure S7B: label of the y axis

Figure S8: move information in the chart title to the legend

**Author's Response to Decision Letter for (FEMSMC-2022-028)**

LptD depletion disrupts morphological homeostasis and rewires carbohydrate metabolism of Escherichia coli

Reviewer: 1

Comments to the Author

The manuscript by Svanberg Frisinger and co-workers reports on the construction and characterization of a CRISPRi strain in E. coli that allows the tuneable expression of the essential lptD gene. In this work, after confirming the appearance of envelope biogenesis-related phenotypes in the strain under induction of the repression system, the authors analyse the changes in the transcription profile upon LptD depletion. From this analysis the authors identify the upregulation of colanic acid biosynthesis genes as the strongest effect of LptD depletion, that they interpret as a mean of the cells to fortify the damaged envelope. Finally, from the analysis of the combination of LptD depletion and colistin treatment the authors observe an even stronger increase in colanic acid biosynthesis gene expression associated to the downregulation of flagella synthesis and chemotaxis genes, indicative of a switch from planktonic to biofilm growth mode. This latter finding has important implications in bacterial virulence.

The overall work is interesting for the readership of the journal, and the topic is relevant. The construction of the LptD depletion strain is a valuable tool to assess the synergy between drug treatments and outer membrane damage. However, the analysis of the transcriptomic data is sometimes too much speculative and lacks appropriate validations to support the main conclusions of the study.

Following I report some comments/suggestions I believe that could help making the manuscript clearer and strengthening the conclusions of the work:

We thank the reviewer for their comments and feedback.

1) The first part of the work is focused on the adaptation of the CRISPRi system to allow the knockdown of lptD, with the aim of identifying the suitable concentration of the inducer that leads to gene repression avoiding the appearance of mutations that inactivate the repression system. The analysis is thorough, but I think that it would be useful to accompany the transcriptional analysis with the analysis of the steady-state level of the LptD protein by western blot under different CRISPRi induction conditions. This would help understanding the degree of protein depletion obtained.

We agree that western blot analysis would add valuable information. However, the project grant that supported the PhD research presented in the current study has unfortunately ended, and we are thus unable to perform any additional wet-lab experiments. Additionally, western blots are semi-quantitative measurements and like RT-qPCR, would require a control to normalize against and therefore also result in relative measurements of protein levels.

2) After the initial characterization, the authors test whether the knockdown strain displays the known phenotypes associated to LptD depletion and therefore assess cell viability and cell morphology using light microscopy and TEM. The morphological analysis using full (1 mM) and mild (0,2 mM) induction conditions at different time points shows the appearance of a filamentous phenotype of the cells that turns to normal after 24 h and the authors explain this phenomenon with the appearance of suppressor mutations. However, in Fig 2C the viability of the cells treated with 0.2 mM IPTG seems not to be restored at 24 h post induction. Could the authors please clarify on this?

A discussion on this discrepancy has been added (lines 220-226).

3) At line 172, the authors suggest that the reduction in the size of the periplasm, visible in Fig. 3C, is indicative of LPS accumulation. I suggest the authors indicate the reference for this statement. The accumulation of LPS in the inner membrane is also mentioned as responsible of the downregulation of lpxK gene expression at line 206, however LPS accumulation in the inner membrane under these conditions is not confirmed experimentally. I think that the authors should demonstrate the LPS accumulation in the inner membrane by western blot using anti LPS antibody on inner and outer membrane fractions.

A reference has been added to support the statement (lines 229-230). As mentioned before, this PhD project ended and performing any further experiments will not be possible (see answer to comment 1).

4) In the paragraph starting from line 181, the authors mention 3 genes (pdxA, prpR and yahO) that are differentially regulated compared to the uninduced conditions under both the induction conditions analysed (0.1 and 1 mM IPTG), but no comments are made on this, and the relevance of this data is not clear. Could the authors please comment on this?

Done (lines 251-259).

5) The synergy between lptD knockdown and colistin treatment is interesting and the RNAseq analysis suggests that the combination of the two conditions is associated to the upregulation of colanic acid biosynthesis genes. The authors suggest that this might be a strategy used by the cell to fortify the cell envelope. Also in this case, the authors should strengthen their hypothesis by quantifying the level of colanic acid produced under the conditions tested and possibly by evaluating the sensitivity to colistin of a mutant deleted for wcaJ (that is therefore not able to produce colonic acid) under lptD knockdown.

We agree with the reviewer that it would be interesting but unfortunately performing any further experiments will not be possible (see answer to comment 1).

Minor points:

Figure 1A: The transmembrane helix of LptC should be inserted between LptF and LptG subunits.

Answer: Done (line 103)

Figure 2A: the authors state that the growth profile shows a dose-dependent response from 0,15 mM IPTG but I think that the effect is dose-dependent only at concentration ≥ 0.17 mM

This has been corrected and updated throughout the manuscript.

Figure 3C: It would be useful to indicate in the low magnification image where the markers of cell deterioration that are zoomed in in the panels on the right are located

The images are taken of different cells and it is therefore not possible to make that reference.

Line 207: lipid IV A derives from LpxK-mediated phosphorylation of the precursor disaccharide-1-phosphatedisaccharide, please correct.

Done (lines 274-275).

Figure S8 should show the synergy between colistin treatment and LptD depletion, however the graph is not clear to me, and it seems that a panel is missing.

The correct graph has been inserted together with an updated figure legend (see Supplementary Materials).

Reviewer: 2

Comments to the Author

The authors examined the effect of the lptD knockdown by using CRISPRi. The experimental design is good, but the presentation of figures are too small to understand. I cannot see whether there is a difference or not in Figures 4 and 5. Also, statistical analysis should be performed to support the author's conclusion.

We thank the reviewer for their comments and feedback. Both figure 4 and 5 are updated with larger size image and font. The statistical analysis has been referenced in the manuscript and is further expanded on in questions 4 and 7 below.

Main concerns

1) Fig S1, explanations for NC_1360, NC_1212, NC163, and NC_118 are needed.

The figure legend has been updated to make the figure clearer (see Supplementary Materials).

2) Fig. 2, growth curves were probably obtained by measuring OD600 in microplate reader. The amount of broth in the well, temperature and shaking conditions, and microplate reader product name should be described.

The requested information has been added to the materials and methods (lines 515-517).

3) Lines 395-401, oligonucleotide primer sequences should be presented. What gene was used for internal control? The internal control gene also should be described in the legend of Figure 2B.

Answer: Done (lines 551-554 and 204).

4) Figures 4 and 5, the size is too small to understand the data. I cannot see the labels for the axis. In addition, is statistical analysis performed? Statistical method and the values should be presented in the legends or in the figures.

Answer: Figures are updated with larger size image and font (lines 285 and 339). All the statistical analysis of RNAseq data were performed following standard DESeq2 analysis pipeline. The reference of DESeq2 method is included in the revised manuscript (line 563). p-values of individual log2FC datapoints are presented in the supplemental data (Supplementary tables 1 and 2).

5) Figure S5, I cannot see the labels for the axis.

A larger version with better resolution has been made (see Supplementary Materials).

6)Figure S7A, what is the closed circles and the small dots? These should be explained in the legends.

Done (see Supplementary Materials).

7) Figure S7B, What gene was used for internal control? Was Statistical analysis performed?

In DESeq2 RNAseq data analysis pipeline, sequence read counts for each gene are modelled following a generalized linear model (GLM). The GLM fit return matrix coefficient identify the log2FC between the two groups of samples.

Reviewer: 3

Comments to the Author

This article by Frisinger et al. reports effects of the lptD depletion in E. coli by the CRISPRi method, including defects in cell morphology and cell growth, as well as expression pattern change of genes in carbohydrate metabolism. The authors also introduced colistin treatment to the lptD-depletion strain and identified up- or down-regulation of motility, chemotaxis, and virulence genes. Although the CRISPRi method appears simple and fast, there are some concerns needing to be clarified before interpreting the results.

We thank the reviewer for their comments and feedback.

1. Modification of the title “LptD depletion disrupts morphological homeostasis and rewires carbohydrate metabolism of Escherichia coli” is recommended, since this work does not directly measure the carbohydrate metabolism. Instead, gene expression is investigated.

We thank the reviewer for bringing this to our attention. We modified the title to - "LptD depletion disrupts morphological homeostasis and upregulates carbohydrate metabolism in Escherichia coli”

2. Since the morphological defect was documented in Braun & Silhavy (2002), the author please discuss any difference in morphology between the previous and current studies using different methods.

The indicated reference has been added to the discussion on the discrepancy in morphology between different studies (lines 363-370).

3. The essential details of the CRISPRi experiments are missing.

What is the sequence of sgRNA? The cited reference of Wang et al. 2018 (line 355-6) reported 15 sgRNAs of lptD (Supplementary Data 3 and 4) and the experiment was performed with pooled sgRNAs. Which one is used in this study? How did the author select the particular sgRNA?

How were the RT-qPCR data quantified?

How was the reported fold change of gene expression defined and calculated?

What is the reference gene used in quantification?

The materials and methods have been updated with a table of the various sgRNA sequences used throughout the study, and the selection process has been described (lines 483-484 and 493).

The materials and methods have been updated to include more details on the RT-qPCR methodology (lines 544-554).

4. The authors please address how do the drawbacks of the CRISPRi method impact this study, which include off-target, bad-seed, and polar effects.

The increasing IPTG concentration to change the expression level of dCas9 is to examine the bad-seed effect. Judging from Figure 2C and S4, IPTG at 0.2 mM could be more than sufficient to balance cell viability and morphological defect.

The off-target effect of the sgRNA used in the study needs to be discussed in detail.

There are also adjacent genes up- and downstream to lptD. Whether the polar effect occurs along with the lptD depletion needs to be examined in order to clarify whether or not any polar effect influences the observations.

A section discussing the abovementioned drawbacks and their implications has been added to the discussion (lines 430-445).

5. Because of the possible bad-seed effect with dCas9 at high concentration, those experiments using 1 mM IPTG induction may not be relevant to lptD depletion.

Line 186-187: It is questionable about the relevance of the genes identified using 1 mM IPTG. Same for Figure 3C: TEM data

The sgRNA sequence used to target lptD was selected as the seed sequence has been shown to show no off-target effect, and the particular seed sequence is not associated with a strong ‘bad-seed’ effect (Cui et al., 2018). Thus we would like to argue that the experiments performed at 1mM IPTG induction are still of relevance and interest.

6. The statement for the colistin treatment on top of the lptD depletion is arguable. Since the complex is disrupted in the lptD-depletion strain, the effects seen after colistin treatment do not go through the lpt protein complex. It is more likely that the lptD depletion reduces OM integrity and the colistin treatment resulting in cell surface damage is an “additive”, instead of “synergistic”, effect. Both lptD depletion and colistin treatment can trigger stress responses in bacterial cells, but likely through different mechanisms.

We agree that both LptD depletion and colistin treatment lead to different effects on the cell. However, we show that the low levels of lptD depletion do not modulate the transcriptome of E. coli significantly, and the impact of sublethal concentrations of colistin is very mild on the transcriptome. However, the low levels of lptD depletion together with sublethal concentrations of colistin generate a very strong response in the cell. Thus, the effect of the combination treatment is not equal to the sum of the single agent treatments, indicating a synergistic effect.

7. Following point 3, the up- or down-regulated genes associating with addition of colistin in the lptD-depletion strain relates to colistin, but not necessarily related to the lptD depletion.

In line with the response to the previous comment, we have performed analyses on both colistin treatment and lptD depletion alone and in combination, and have found that the impact of colistin is very strong only under the partial depletion of lptD. We would therefore like to argue that this response is due to the combination of colistin exposure and lptD depletion.

8. The sgRNA is integrated in the chromosome down stream of glmS (line 359) that encodes an enzyme catalyzing the first reaction in the UDP-GlcNAc synthesis pathway. UDP-GlcNAc is the precursor molecule of LPS and PG. Since Figure 4CE and S7A show changes in nucleotide sugar synthesis, the authors please comment on whether integration of the sgRNA plasmid at this chromosomal location influence glmS expression or not.

Answer: The Mobile-CRISPRi system is integrated downstream of glmS and does not disrupt the gene function (Peters, J.M., Koo, BM., Patino, R. et al. Enabling genetic analysis of diverse bacteria with Mobile-CRISPRi. Nat Microbiol 4, 244–250 (2019). https://doi.org/10.1038/s41564-018-0327-z). This information has been added to the materials and methods (lines 489-491).

Other comments

9. Need to provide strain, plasmid, and primer tables.

Done (lines 479, 492 and 509).

10. Since LptD is a low-abundance protein (line 276-277, 60 molecules per cell), the wording of "high-fold depletion" is confusing.

The wording has been changed (line 351).

11. Labels are too small in most figures.

Figures have been modified with larger image and font size.

12. “suppressor” mutant instead of “escape” mutant (line 137, 170, Figure S3)

Done (lines 159, 168, 170 and Supplementary Materials).

Line 59: periplasmic

Done (line 63)

Line 70: terminus

Done (line 75)

Line 183: Is the level of leaky expression determined under the un-induced condition?

No leaky expression was detected in the RT-qPCR experiment (Fig 2B).

Line 189: pdxA is one of the downstream genes to lptD. Address polar effect or not.

Done (lines 252-259).

Line 230: How does the growth reduction affect the observation?

Although growth was reduced in the presence of colistin and IPTG combination, the OD of the culture increased from 0.05 to 2 (4 fold). So, the transcriptional response observed here is likely from the cells growing under the stress.

Line 274: high-fold knockdown?

Done (line 351)

Line 275: “slow” protein turnover

Done (line 352)

Line 277: 60 molecules per cell: Is it possible to estimate a number from the RT-qPCR data?

Unfortunately this is not possible as RT-qPCR only indicates the level of mRNA present in the cell compared to a reference, and does not directly provide information on the number of molecules.

Line 293: What is the non-coding control? With or without sgRNA? Or with or without induction of dCas9?

The text has been revised to address this comment (lines 371-372) and the use of controls has been further explained in the results (lines 127-130)

Materials and Methods

Lines 356-361: contain redundant information.

Done (lines 481-491).

Line 364-: Alternatively, the cell viability can be easily tested by spotting on agar plates.

This experiment has been performed, and the results are presented in Fig 2C (line 172).

Line 395: Details of the RT-qPCR experiment are necessary.

The section has been revised providing details on the primer sequences and the control gene (lines 544-554).

Figure 1: (1) all symbols indicating different components need to be labeled and explained, including the outer and inner membranes. (2) (B) is a generic illustration of the CRISPRi system and does not provide specific information to lptD knock-down in this work. This figure needs to include the sequence of sgRNA, its priming site, and the location of where it is integrated on chromosome, etc.

Done (line 103).

Figure 2: need experimental details to validate 2B. Correct label of the y axis.

Done (line 172 and lines 544-554).

Figure 3: (1) The scale bar size in micrographs is not correct. (2) Presenting micrographs in (B) and (C) in the same scale is recommended. (3) In (C), use arrows or symbols to indicate features that correspond to the morphological defects or LPS accumulation, etc.

(1) We can’t address this comment because we don’t understand what is incorrect in the scale bar size. (2) The images were selected to be able to show both an overview of the whole cell shape as well as zoomed-in details and we were thus unable to present them with the same scale. (3) Done (line 235).

Figure 4: Too small to see.

The figure has been modified with a larger size image and font (line 285).

Figure 5. Please comment on whether the growth defect considered in the experimental design or in the analysis. Too small to see.

The RNAseq data analysis was performed following DESeq2 pipeline where growth defect gets normalized. The figure has been modified with a larger size image and font (line 339).

Figure S4: missing size of scale bar

Done (see Supplementary Materials).

Figure S5: too small to see

Done (see Supplementary Materials).

Figure S7B: label of the y axis

Done (see Supplementary Materials).

Figure S8: move information in the chart title to the legend

Done (see Supplementary Materials).

**Reviews of manuscript FEMSMC-2022-028.r1**

**Reviewer 4**

**Recommendation: Major Revision**

In this manuscript, the authors applied CRISPRi to deplete lptD in E. coli and characterized the morphological phenotypes and transcriptional responses. Minor comments from the former reviewers were adequately addressed. However, the authors did not address some of the major points raised by former reviewers 1 (e.g. LptD protein levels, LPS accumulation in the IM, and colonic acid levels) and 3 (e.g. impact of polar effects), citing inability to conduct additional experiments as the project/grant has ended. This reviewer thought the reason for not doing more experiments (to make the manuscript more robust) seems weird, as one would assume the PI and collaborators should have other funding in their groups to facilitate the simple experiments suggested. In any case, this reviewer has the following comments that the authors should look into.

1. Based on the methods, it appears that the transcriptomics data was only obtained for single samples from each condition (no replicates at all). This may explain why there are no error bars included in the data presented in the figures. This reviewer thinks that transcriptomics without replicates might not provide data sufficiently robust for interpretation. In this case, the reviewer would like to have seen RT-PCR validation of the initial transcriptomics before the authors go about making sense of the apparent upregulation of carbohydrate metabolic pathways and colanic acid biosynthesis.

2. The impacted of off-target and polar effects of CRISPRi using the lptD sgRNA have not been tested (similar to former reviewer 3 point 4). In terms of off-target effects, the authors could have use CRISPRi to knockdown other lpt genes, which should in principle phenocopy lptD knockdown. In terms of polar effects, while the authors mentioned that the levels of other downstream genes besides pdxA were not significantly altered, the transcriptomics data may not be sufficiently robust to make such claims (see point 1). The authors should use RT-PCR to show no impact on downsteam genes, especially for surA, which would have cause major envelope stresses when deleted/depleted.

3. Lines 214-216: How did the authors measure cytoplasmic density and the width of the periplasm? There is no data to support these claims.

4. Lines 258-260: “Moreover, intracellular accumulation of LPS was found to lead to the downregulation of lpxK…” While the authors do not wish to experimentally measure if CRISPRi knockdown of lptD causes LPS accumulation in the IM (as seen in former reviewer 1 point 3), they used this claim to explain why they might have seen downregulation of lpxK. This reviewer thinks LPS accumulation in the IM should be shown experimentally, perhaps by looking at build-up of LPS linked to colanic acids.

5. Lines 256-258: “Since the O-antigen of E. coli LPS is fucosylated (Yi et al., 2009), the up-regulation of GDP-sugar metabolism might be important to compensate for its reduced levels in the extracellular environment.” The authors used BW25113 in this manuscript, which does not make O-antigens due to an IS insertion in wbbL. In addition, the O-antigen made by BW25113 would be the O-16 serotype, which does not contain fucose, unlike the O-86 serotype in cells used in the Yi et al 2009 cited paper. Therefore, this reasoning for why GDP-sugar metabolism may be upregulated is flawed.

6. Is the upregulation of colanic acids dependent on the rcs phosphorelay pathway?

**Reviewer 1**

**Recommendation: Accept**

I thank the authors for responding to the issues I raised in my initial review. I have no further questions and find the manuscipt suitable for publication.

I have only a minor comment about the revised part of the manuscript:

-line 124: the authors state that "At concentrations ≥ 0.17 mM growth was similar to that of the uninduced control,...", but I think that they intended ≤ 0.17 mM.

**Reviewer 2**

**Recommendation: Accept**

The authors have adequately addressed my concerns.

**Editor’s notes to author**

The manuscript describes a CRISPRi strain's response to about 10 levels of induction on growth, phenotype (via TEM), and transcript levels of the gene targeted by the CRISPRi (via qRT-PCR, with replicates). All of that is good.

The third reviewer raises good points about the RNASeq data. For the RNASeq, the manuscript isn't clear what the sample sizes are, and the exact structure of the experiment (3 induction levels were used, plus the addition of an antibiotic...methods not clear on how this was mapped out (6 samples? More?)).

Please address the third reviewers' comments and clarify the details of the RNASeq experiment. If there is a single RNASeq replicate for each condition, it would be great to have follow-up qRT-PCR to confirm transcript abundance changes in a couple of target genes key to their discussion (one gene each from motility and colanic acid metabolism). If however RNASeq n >1, the qRTPCR is less critical to the quality of the work.

**Author's Response to Decision Letter for (FEMSMC-2022-028.r1)**

Associate Editor's Comments to Author/s:

The manuscript describes a CRISPRi strain's response to about 10 levels of induction on growth, phenotype (via TEM), and transcript levels of the gene targeted by the CRISPRi (via qRT-PCR, with replicates). All of that is good.

The third reviewer raises good points about the RNASeq data. For the RNASeq, the manuscript isn't clear what the sample sizes are, and the exact structure of the experiment (3 induction levels were used, plus the addition of an antibiotic...methods not clear on how this was mapped out (6 samples? More?)).

Please address the third reviewers' comments and clarify the details of the RNASeq experiment. If there is a single RNASeq replicate for each condition, it would be great to have follow-up qRT-PCR to confirm transcript abundance changes in a couple of target genes key to their discussion (one gene each from motility and colanic acid metabolism). If however RNASeq n >1, the qRTPCR is less critical to the quality of the work.

We thank the editor for their comment. In the RNA Seq experiment, we used 7 different conditions (6 experimental and 1 control) and maintained 4 separate replicates for each condition totalling 28 samples. Thus, we consider the experiment is statistically robust. The materials and methods have been updated in order to clarify this (lines 464-467).

Reviewers’ Comments to Author/s:

Reviewer: 4

Comments to the Author

In this manuscript, the authors applied CRISPRi to deplete lptD in E. coli and characterized the morphological phenotypes and transcriptional responses. Minor comments from the former reviewers were adequately addressed. However, the authors did not address some of the major points raised by former reviewers 1 (e.g. LptD protein levels, LPS accumulation in the IM, and colonic acid levels) and 3 (e.g. impact of polar effects), citing inability to conduct additional experiments as the project/grant has ended. This reviewer thought the reason for not doing more experiments (to make the manuscript more robust) seems weird, as one would assume the PI and collaborators should have other funding in their groups to facilitate the simple experiments suggested. In any case, this reviewer has the following comments that the authors should look into.

1. Based on the methods, it appears that the transcriptomics data was only obtained for single samples from each condition (no replicates at all). This may explain why there are no error bars included in the data presented in the figures. This reviewer thinks that transcriptomics without replicates might not provide data sufficiently robust for interpretation. In this case, the reviewer would like to have seen RT-PCR validation of the initial transcriptomics before the authors go about making sense of the apparent upregulation of carbohydrate metabolic pathways and colanic acid biosynthesis.

We thank the reviewer for their comments and feedback. In the RNA-Seq experiment, 7 different conditions were used each based on 4 separate replicates, and the materials and methods have been updated in order to make this clearer (lines 464-467). Fig 4A, C, E & Fig 5A all lack error bars as the smaller dots represent the individual genes within the systems displayed and the larger dots the average expression change of the system (explained in the figure legends (lines 229-232 and 283-285)), and we believe that adding error bars would make the graphs more complex and less easily interpretable.

2. The impacted of off-target and polar effects of CRISPRi using the lptD sgRNA have not been tested (similar to former reviewer 3 point 4). In terms of off-target effects, the authors could have use CRISPRi to knockdown other lpt genes, which should in principle phenocopy lptD knockdown. In terms of polar effects, while the authors mentioned that the levels of other downstream genes besides pdxA were not significantly altered, the transcriptomics data may not be sufficiently robust to make such claims (see point 1). The authors should use RT-PCR to show no impact on downsteam genes, especially for surA, which would have cause major envelope stresses when deleted/depleted.

We have previously developed other CRISPRi mutants targeting lptE but were unable to generate a phenocopy version, which indicates that this may not be an ideal method to verify the lptD depletion. However, the observed morphology presented here is generally well in line with previous observations of LptD depletion (Braun, M., & Silhavy, T. J. (2002). Imp/OstA is required for cell envelope biogenesis in Escherichia coli. Mol Microbiol, 45(5), 1289-1302). As explained under point 1, each RNAseq experiment is based on four individual replicates which should provide enough statistical robustness to be reliable. As we did not see any significant changes in the expression of surA in this RNAseq experiment we conclude that this and other genes situated downstream were not significantly affected by our lptD-knockdown. This has also been included in the discussion (lines 364-371).

3. Lines 214-216: How did the authors measure cytoplasmic density and the width of the periplasm? There is no data to support these claims.

Neither the cytoplasmic density nor the width of the periplasm were measured or discussed in the manuscript. We only reported and discussed morphological changes of the cells observed using light and transmission electron. In order to avoid confusion for the reader, we have updated the text to make this clearer (line 181).

4. Lines 258-260: “Moreover, intracellular accumulation of LPS was found to lead to the downregulation of lpxK…” While the authors do not wish to experimentally measure if CRISPRi knockdown of lptD causes LPS accumulation in the IM (as seen in former reviewer 1 point 3), they used this claim to explain why they might have seen downregulation of lpxK. This reviewer thinks LPS accumulation in the IM should be shown experimentally, perhaps by looking at build-up of LPS linked to colanic acids.

We agree that measuring whether lptD causes LPS accumulation would be relevant but, as previously explained, we are unfortunately unable to perform this additional work. We have however addressed this comment in the revised manuscript by highlighting the lack of this experimental work and other limitations of this study (lines 389-393).

5. Lines 256-258: “Since the O-antigen of E. coli LPS is fucosylated (Yi et al., 2009), the up-regulation of GDP-sugar metabolism might be important to compensate for its reduced levels in the extracellular environment.” The authors used BW25113 in this manuscript, which does not make O-antigens due to an IS insertion in wbbL. In addition, the O-antigen made by BW25113 would be the O-16 serotype, which does not contain fucose, unlike the O-86 serotype in cells used in the Yi et al 2009 cited paper. Therefore, this reasoning for why GDP-sugar metabolism may be upregulated is flawed.

We thank the reviewer for pointing this out, the statement has now been removed entirely (line 216).

6. Is the upregulation of colanic acids dependent on the rcs phosphorelay pathway?

The regulation of the colanic acid operon is complex, and although it appears to not be completely dependent on the rcs phosphorelay pathway this can positively upregulate the expression of the operon (Stout V. Identification of the promoter region for the colanic acid polysaccharide biosynthetic genes in Escherichia coli K-12. J Bacteriol. & Rahn A, Whitfield C. Transcriptional organization and regulation of the Escherichia coli K30 group 1 capsule biosynthesis (cps) gene cluster. Mol Microbiol.). This has been added to the discussion (lines 350-352).

Reviewer: 1

Comments to the Author

I thank the authors for responding to the issues I raised in my initial review. I have no further questions and find the manuscipt suitable for publication.

I have only a minor comment about the revised part of the manuscript:

-line 124: the authors state that "At concentrations ≥ 0.17 mM growth was similar to that of the uninduced control,...", but I think that they intended ≤ 0.17 mM.

We thank the reviewer for their comment, which is now addressed in the text (line 113).

Reviewer: 2

Comments to the Author

The authors have adequately addressed my concerns.

**Reviews of manuscript FEMSMC-2022-028.r2**

**Reviewer 2**

**Recommendation: Accept**

The authors adequately addressed my concerns.

**Reviewer 1**

**Recommendation: Accept**

After reading the the new version of the manuscript and the comments from Authors, I consider the manuscript acceptable for publication.
